# Neovaginal Prolapse in Male-to-Female Transsexuals: An 18-Year-Long Experience

**DOI:** 10.1155/2014/240761

**Published:** 2014-05-07

**Authors:** Stefano Bucci, Giorgio Mazzon, Giovanni Liguori, Renata Napoli, Nicola Pavan, Susanna Bormioli, Giangiacomo Ollandini, Bernardino De Concilio, Carlo Trombetta

**Affiliations:** Department of Urology, Cattinara Hospital, University of Trieste, Strada di Fiume 447, 34149 Trieste, Italy

## Abstract

Neovaginal prolapse is a rare and distressing complication after male-to-female sexual reassignment surgery. We retrospectively analysed the prevalence of partial and total neo-vaginal prolapses after sexual reassignment surgery in our institute. During the years, two different techniques have been adopted with the aim of fixing the neovaginal cylinder. In the first, two absorbable sutures are placed at the top of the penoscrotal cylinder and fixed to the Denonvilliers fascia. In the second, two additional sutures are added from the posterior/midpoint of the flap to the prerectal fascia. We enrolled 282 consecutive transsexual patients. 65 (23.04%) out of the 282 were treated with the first technique and the following 217 (76.96%) with the last technique. In the first technique, 1 case (1.53%) of total prolapse and 7 cases (10.76%) of partial prolapse were observed, while in the other 217 patients treated with the second technique only 9 cases of partial prolapse were observed (4.14%) and no cases of total prolapse. All prolapses occurred within 6 months from the procedure. In our experience, the use of 4 stitches and a more proximal positioning of the sutures to fix the penoscrotal apex with the Denonvilliers fascia guarantees a lower risk of prolapse.

## 1. Introduction

The final goal of androginoid sex reassignment surgery (SRS) is the creation of a feminine, functional and well-vascularised perinea-genital complex, free of poorly healed areas, scars, and neuromas. Ideally, the neovagina should be 10 cm in depth and about 30 mm in diameter. Moreover, it should be fashioned with moist, elastic, and hairless epithelium [1].

For decades, several techniques have been proposed, but, as suggested by Sutcliffe et al. in a systematic review, no operative standards of care are available in this particular surgical field [2].

These procedures expose patients to several possible early and late complications, leading to loss of aesthetic and functional satisfaction.

Specifically, neovaginal prolapse after sexual reassignment surgery in male-to-female transsexuals is a distressing complication for both patient and surgeon, leading to bad aesthetic and functional outcomes and can sometimes be difficult to correct. The frequency of this complication is difficult to ascertain, and literature only reports single cases (since the anatomic circumstances preceding the operation and the postoperative course are often not known).

Several authors have reported their outcomes after SRS but all of them enrolled a low number of patients, so the real incidence of neovaginal prolapse is not well known.

Perovic et al. in 89 consecutive transsexual male-to-female patients using penile skin and urethral flap had no reported cases of neovaginal prolapse [3].

Similarly, Krege et al. reported 2 cases of prolapse out of 66 patients who had undergone male-to-female SRS by penoscrotal flap vaginoplasty. However, authors did not specify if the prolapses were partial or total [4].

Finally, Djordjevic et al. [5] reported a series of 86 consecutive rectosigmoid vaginoplasties. In their experience 7 cases (8.1%) of partial vaginal prolapse were observed. However, this series comprehends both transsexual patients as well as females affected by vaginal agenesia or who had undergone vaginectomies for genital trauma. All vaginal prolapses were repaired by minor surgery.

We herein report the incidence in our experience of total and partial neovaginal prolapse, how we prevent it, and what the optimal way to correct it is.

## 2. Materials and Methods

**Figure 1:**
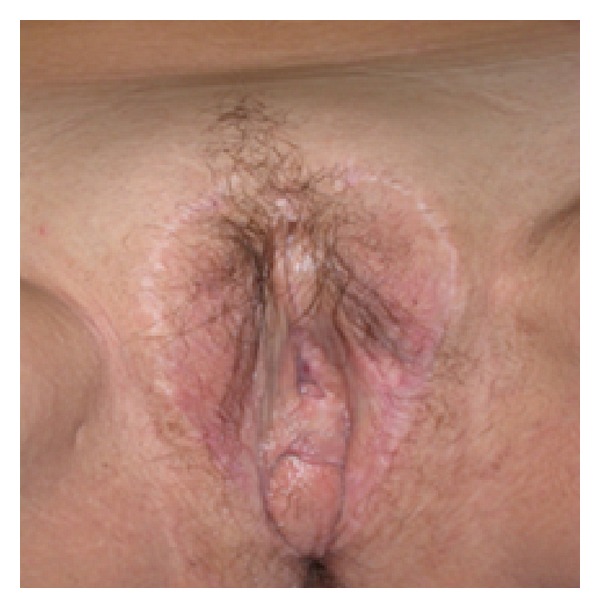
Partial neovaginal prolapse.

**Figure 2:**
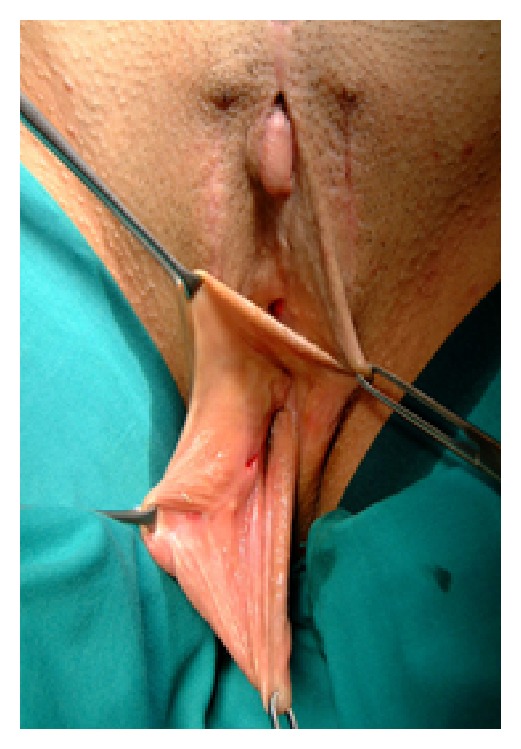
Total neovaginal prolapse.

We retrospectively analysed the prevalence of partial (Figure 1) and total (Figure 2) neovaginal prolapses after androginoid sexual reassignment surgery between December 1994 and January 2012 in our institute. Our procedure includes bilateral orchiectomy, removal of corpora cavernosa, creation of the urethrostomy, neovaginoplasty, and creation of neoclitoris with preservation of neurovascular bundles and neovulvoplasty. Since the end of 2010 we have adopted an original technique, which consists of creating a neoclitoris embedded in urethral mucosa using a urethral flap [6]. In the refinement, the urethra is carefully dissected from the corpora cavernosa within buck's fascia and shortened approximately 7 cm distally from the bulbs. It is then spatulated on its ventral side down to the bulbs where a neomeatus is then created at the level of the female type urethra [4].

**Figure 3:**
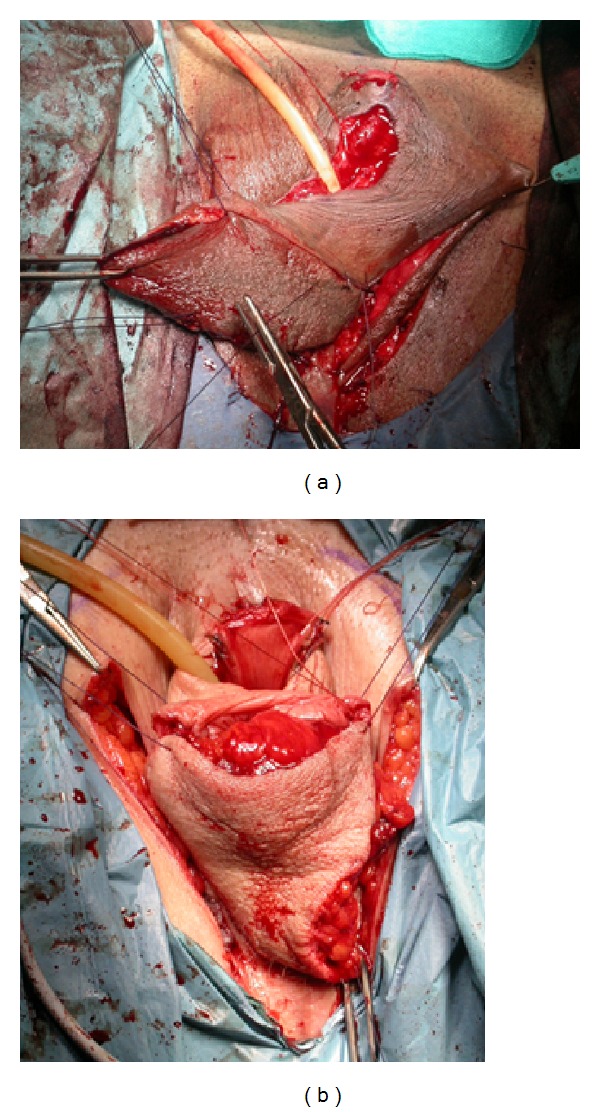
(a) A penoscrotal cylinder is fashioned. (b) At the apex of penoscrotal cylinder two reabsorbable stitches (∗) are positioned, which subsequently will be fixed onto the Denonvilliers fascia.

To create the neovagina, we adopted the penile and scrotal skin inversion technique (Figures 3(a) and 3(b)). We prefer not to close the apex of the neovaginal cylinder; in this way the penile and scrotal skin spontaneously covers the cavity where the cylinder is located, ensuring a deeper neovagina.

During the years, two different techniques have been adopted with the aim of fixing the neovaginal cylinders.

**Figure 4:**
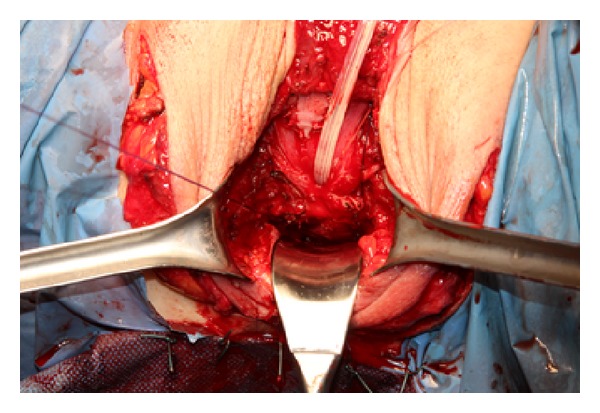
Penoscrotal cylinder is inverted and fixed to the Denonvilliers fascia.

**Figure 5:**
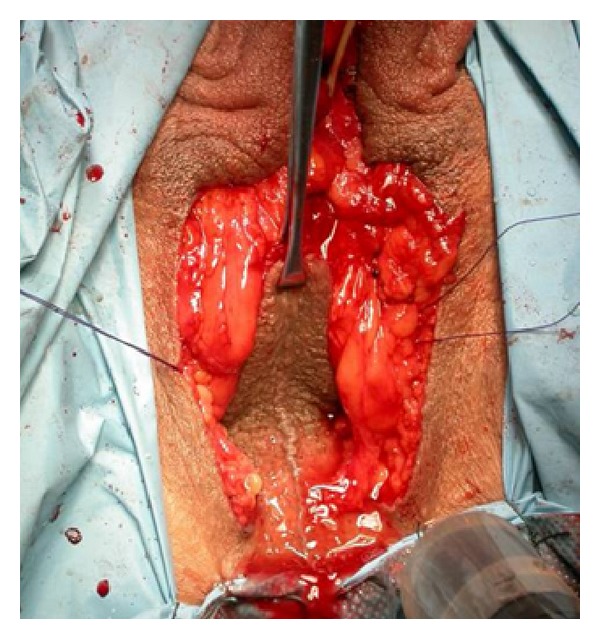
The cylinder is inverted and fixed on its midpart to the prerectal fascia.

In the first, two absorbable stitches (Vicryl 3/0, which requires 35 days to be absorbed) are positioned at the top of the penoscrotal cylinder with the aim of fixing it to the Denonvilliers fascia (2 stitches technique, Figure 4). In the second technique we decided to fix the neovagina with four sutures: two absorbable stitches are fixed from the top of the penoscrotal cylinder to the Denonvilliers fascia and other two from the posterior/midpart of the scrotal flap (which will constitute the posterior neovaginal wall) to the prerectal fascia (4 stitches technique, Figure 5).

When the suture is passed through the Denonvilliers fascia, we often decide to incorporate in the suture some prostatic tissue or seminal vesicles, with the aim of strengthening the sutures.

**Figure 6:**
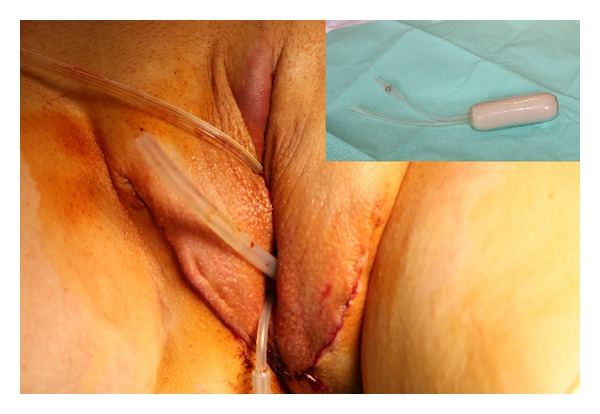
At the end of the procedure a Coloplast vaginal stent is placed inside the neovagina.

**Figure 7:**
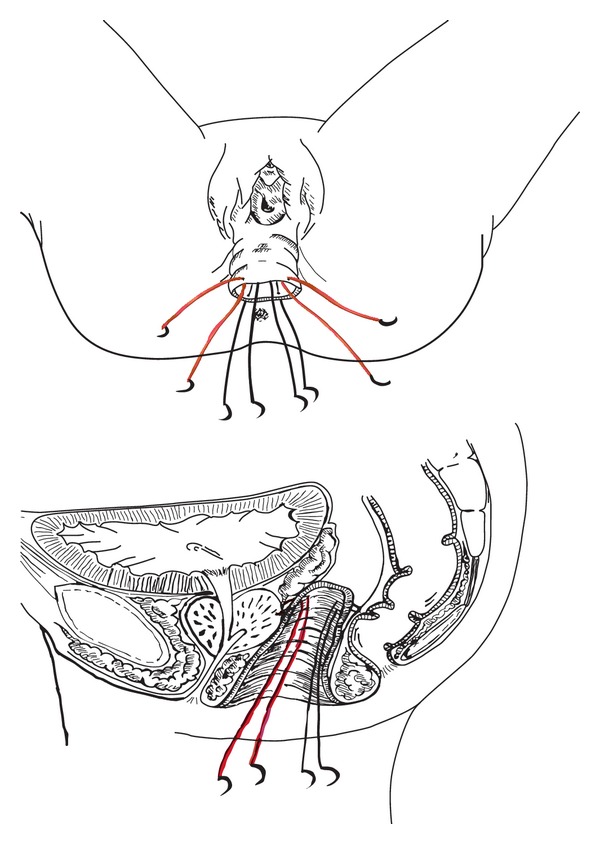
Diagram of where stitches have to be placed, anterior stitches in red and posterior in black.

At the end of the procedure, an inflatable silicon vaginal stent is introduced in the neovaginal cavity where it is maintained both day and night for 3 days, and afterwards only during night-time for a total of three months (Figure 6). We prefer to use a Coloplast (Minneapolis, USA) vaginal stent. This guarantees that the penoscrotal flap will adhere to the cavity, facilitating the recovery and at the same time reducing the risk of stenosis. After 4 days from the procedure, patients are educated by a specialized nurse as to how to self-dilate the neovagina with progressively larger dilators. Neovaginal self-dilation is a fundamental step for a good long term result, first of all for maintaining the depth of the neovagina but also for prevention of vaginal prolapse. Patients must learn how to perform the dilations well, without stretching the penoscrotal flap. In Figure 7, a scheme of the procedure is reported.

Patients are systematically reevaluated at 6 and at 12 months after the procedure.

Statistical analyses were performed with SPSS 17.0 software. We compared median values using *t*-test, if appropriate, or Wilcoxon sign-rank test. *P* values <0.05 were considered significant.

## 3. Results

282 consecutive male transsexuals who had undergone male-to-female sex reassignment surgery (SRS) at our institute were enrolled. 65 (23.04%) out of the 282, were treated with “two stitches” technique and the following 217 (76.96%) with the “four stitches” technique. Out of all of our patients, the first 9 were operated on using the inverted penile skin vaginoplasty approach, whereas in the other 273 a penile and scrotal skin inversion technique was used.

Out of the 65 patients operated with the “two stitches” technique, 8 patients presented a neovaginal prolapse (12.30%).

1 case (1.53%) of total prolapse and 7 cases (10.76%) of partial prolapse were observed, while in the other 217 patients treated with “four stitches” technique only 9 cases of partial prolapse were observed (4.14%) and no cases of total prolapse. Considering partial prolapses, 10 occurred at the posterior vault and 6 at the lateral vault. All prolapses occurred within 6 months from the procedure. Results are reported in Table 1; differences between groups are statistically significant, except for total prolapse (*P* = 0.225).

## 4. Discussion

In our data, only one patient developed a total neovaginal prolapse. In this case, the “two stitches” technique was used. Moreover, the “two stitches” technique seemed to more frequently determine a partial prolapse in comparison to the “four stitches” technique (10.76% and 4.14%, resp.).

Penoscrotal flap vaginoplasty is one of the most common surgical procedures adopted nowadays to create a neovagina in male-to-female transsexuals.

Different methods for suspension of the neovagina have been described.

Stanojevic et al. proposed sacrospinous ligament fixation of the neovaginal wall to prevent prolapse. Authors have not preferred prolapse after 62 consecutive patients were treated with this technique [7]. We prefer not to use this procedure because it requires extreme caution in consideration of the anatomic relationship to the pudendal vessels and nerves, sciatic nerve, ureter and rectum.

Other authors propose a nonsuture fixation of the neovagina with pliable lubricated [8] intravaginal packing that is left in place postoperatively for 5 days. However, we consider this technique at high risk of prolapse. In our technique, since using four stitches, the prolapse of the neovaginal vault is exceptionally rare: two are in order to suture the vault to the prostate and two to suture the rectum to the lateral part of the neocavity.

We believe that fixing the apex of the penoscrotal flap to the Denonvilliers fascia avoids the risk of total prolapse, while suturing the midpoint of the cylinder considerably reduces the risk of partial prolapse.

Sacropexy with synthetic mesh should be the most valid approach to the neovaginal prolapse as the correct neovaginal axis is restored and neovaginal function is preserved. This technique guarantees an adequate neovaginal depth and an excellent functional result. The main cause for suspension failure and the detachment of stitches from the neovaginal wall is also reduced not only because of the large vagina-mesh contact area but also thanks to the no-traction suspension. This is possible because the length of the mesh is regulated by the distance between the neovagina and sacral promontory.

Long-term outcomes of prolapse treatment in transsexual patients are not available in literature. A review of the literature including 40 studies published in 2011 provides an update of surgical management of pelvic organ prolapse in women [9]. The first problem is to define what the best surgical choice for prolapse treatment is. Authors compared outcomes of abdominal sacropexy versus vaginal sacrospinous colpopexy. Abdominal sacral colpopexy was better than vaginal sacrospinous colpopexy with a lower rate of recurrent vault prolapse (RR 0.23, 95% CI 0.07 to 0.77) [10, 11], even if associated with longer operating time and even if it is more expensive.

A second problem is if the colpopexy must be performed with absorbable or nonabsorbable grafts. One trial compared abdominal sacral colpopexy using either an absorbable cadaveric fascia lata graft (Tutoplast) or a nonabsorbable monofilament polypropylene mesh (Trelex). In both groups there were no recurrences of vaginal prolapse [12].

To the best of our knowledge, large databases of transsexual patients who underwent colpopexy for neovaginal prolapse do not exist in literature; only single cases are at best reported [13, 14], and in all of these an open approach was used. In reality, in our experience the sacropexy results are difficult in patients who have undergone ileal vaginoplasty because the ileal walls are not easily stretched with respect to the penal scrotal graft that on the other hand results in having a much more malleable and extendable and resistant wall and therefore is more adequate for this type of surgery.

The same surgery has already been described laparoscopically. This procedure was reported for the first time in 2006 [15] with the aim of restoring the neovagina without compromising its function.

The optimal choice for treating partial prolapse is not very clear; however even in these cases colposacropexy is most likely the best choice. In 6 out of the 17 patients affected by partial prolapse we decided to reposition the two sutures in the midpoint of the cylinder, but the risk of recurrence was very high; in fact 4 of them referred a partial prolapse again. In these cases no other surgical procedures have been performed. In the remaining 11 patients no surgical procedure was performed.

We had a single case of total neovaginal prolapse. In this case, considering that she had undergone an abdominal exploration for acute local peritonitis 7 years before, we decided to correct it with an open colposacropexy. Prolapse occurring after several months was caused by the disuse of lubrication during sexual intercourse. In all 3 cases, patients reported the presence of prolapse after prolonged sexual intercourse in “uncomfortable places” without the use of any type of lubricant.

In order to avoid stenosis and prolapse of the neovagina it is very important to use the vaginal stent regularly after surgery. In our opinion, it has several advantages. First of all, the stent maintains an adequate depth and diameter of the neovagina and guarantees that the skin cylinder will adhere to the cavity, facilitating the recovery and, at the same time, reducing the risk of stenosis. Moreover, it assures a good drainage of fluids collected inside the neovagina, reducing the risk of infection.

Our study has some limitations: we have not considered if our technique influences the length of the neovagina, and, moreover, data regarding sexual satisfaction during penetration was not available. Furthermore, being a retrospective analysis, it was not possible to verify when the prolapse occurred after SRS.

However, in our experience, all of the prolapses occurred within 6 months from SRS, and we believe that a crucial role in prolapse prevention is performed postoperatively by patients.

In fact, patients must be adequately informed about the management of their neovagina after surgery. Daily dilations are mandatory in order to maintain depth and avoid stenosis. The use of abundant lubrication, with the aim of reducing friction during dilatations and intercourse, which can cause detachment of the skin cylinder and prolapse, is crucial.

To the best of our knowledge, this is the largest study investigating vaginal prolapse in male-to-female transsexuals after SRS.

## 5. Conclusion

In our experience, a more proximal position of the sutures to fix the penoscrotal apex to the Denonvilliers fascia guarantees a lower risk of prolapse. Specifically, total neovaginal prolapse has no longer been observed and partial prolapse now has a lower incidence.

Positioning 4 suture stitches is a short procedure and guarantees excellent functional outcome.

Moreover we believe that the postoperative management, in particular the early use of the vaginal dilator for self-dilatation and adequate lubrication, is mandatory and as important as timing and compliance of the patients in order to achieve a good aesthetic and functional result.

## Figures and Tables

**Table 1:** Comparison of prolapse prevalence in “two stitches” versus “four stitches” groups.

	Old technique	New technique	*P*
Patients	65 (23.04%)	217 (76.96%)	

No prolapse	57	208	0.026
Prolapse	8 (12.30%)	9 (4.14%)	0.031
Partial	7 (10.76%)	9 (4.14%)	0.019
Total	**1 (1.53%)**	**0 (0%)**	**0.225**
